# Human defensins and Th-1 cytokines in hepatitis C viral infection

**DOI:** 10.11604/pamj.2020.37.103.25211

**Published:** 2020-09-29

**Authors:** Dorcas Ohui Owusu, Michael Owusu, Bright Afriyie Owusu

**Affiliations:** 1 Department of Medical Laboratory Technology, Garden City University College (GCUC), Kumasi, Ghana,; 2 Department of Medical Diagnostics, Kwame Nkrumah University of Science and Technology (KNUST), University Post Office, Kumasi, Ghana

**Keywords:** Hepatitis C, chronic, spontaneous recovery, cytokines, human defensins

## Abstract

**Introduction:**

active or chronic exacerbated forms of hepatitis C virus (HCV) infection subsequently progress to liver disease and human defensins has been determined to have some level of anti-viral properties invitro whilst the expression of T helper-1 cytokines is known to promote complete recovery from acute HCV infection. The study sought to determine relationship between these immune responses.

**Methods:**

a cross sectional descriptive study design was employed. Hundred and thirty-two individuals were assessed were assessed for to anti-HCV, HCV RNA, serum levels of human alpha defensins 1 (HAD-1) and human beta defensins 1 (HBD-1). T helper 1 cytokines (IL-2, IFN gamma, TNF alpha) secreted in serum were also analyzed using commercial ELISA assay. The study was conducted in Kumasi, Obuasi and Daboya in Ghana.

**Results:**

the serum mean concentrations of HAD-1, HBD-1, IL-2, IFN gamma and TNF alpha showed no significant difference in concentrations among participants with chronic, spontaneously recovered or negative to HCV infection (p>0.05). Persons with hepatitis B co-infection were more likely to develop chronic HCV infection (p=0.039). HAD-1 and HBD-1 showed significant positive association with IL-2 (p=0.000) whilst only HAD-1 positively correlated with IL-2 (p<0.000).

**Conclusion:**

the immunological markers determined had no association with the status of HCV infection. HAD-1 increased with increasing levels of IL-2. These findings suggest that during HCV infection, inflammatory response through the production of cytokines by IL-2 cells may affect the release of HAD-1 and HBD-1.

## Introduction

Hepatic viruses including hepatitis C virus (HCV) are the leading cause of liver diseases in Africa. It is estimated that about 220 million people worldwide are infected are infected with HCV [1]. The genome encodes distinct non-structural and structural proteins: core (C) protein, envelope proteins (E1 and E2) and a short hydrophobic peptide (p7) [2]. The outcome of infection with the virus varies from asymptomatic acute or chronic disease to fulminant hepatitis leading to liver diseases and or hepatocellular carcinoma [1]. Currently, there are no vaccines available against HCV and the treatment offered include pegylated interferon (PEG-IFN) and ribavirin in combination with protease inhibitors as well as direct acting anti-virals. But their use is subjected to the patients´ response, side effects, viral resistance and treatment cost [1]. Viral resistance is enhanced by the virus´s ability to form quasi species during replication. These limitations on viral therapy is a major challenge in the management of HCV infection [2]. The body´s own defence mechanisms play an important role in self-limiting HCV infection leading to complete viral clearance without medical intervention. Understanding of the innate immune system in response to viral infection indicates activation of the innate immune system is crucial for subsequent adaptive immune responses [3]. These include the production of specific antibodies, CD4+ T-cell and cytotoxic T-cell activation which play a major role in resolving viral infections.

Some studies have showed up-regulation of T helper 1 (Th-1) cytokine profile is associated with recovery from HCV infection, whilst the production of more T helper 2 (Th-2) cytokine takes place in development of persistent infection [4]. However, HCV is able to evade both the innate and adaptive immune systems in some individuals to sustain an infection the chronic phase. Human defensins, part of the innate immune responses are a group of antimicrobial peptides produced in microbial infections. Even though they are also produced to aid other antimicrobial agents, a few studies have indicated some level of antiviral activities [5]. Defensins are activated by signal transduction and regulation of the inflammatory response. They are however involved in regulating proliferation and release of Th-1 cytokines through the effective regulation of complement activation and degranulation of mast cells [6]. The pathogenesis of HCV infection has also been determined to include increase production of some cytokines and chemokines by liver mast cells as well as complement production and steroid production [7,8]. The likely role played by human alpha and beta defensins in hepatitis C infection is not well understood, thus the current study sought to help understand the relationship and part played by human defensins.

## Methods

**Study design and setting:** the study was of descriptive cross-sectional design. The population for this study was derived from persons who had tested positive for HCV positive using a rapid serological assay test. These individuals were recruited from a previous study at the Komfo Anokye Teaching Hospital, Obuasi Municipality and Daboya community in Ghana. We recalled these individuals using records obtained from the previous work, which was carried out between 2013 and 2014 (unpublished). Komfo Anokye Teaching Hospital (KATH) is the second largest referral medical facility in Ghana. It is located in Kumasi, the capital of Ashanti region in Ghana. It attends to an average of 400,000 outpatient cases and 41,000 in-patients a year. Obuasi Municipality is a big cosmopolitan municipal in Ashanti region with varying intercultural background due to ongoing mining activities. Daboya community is a district capital in the Northern part of Ghana with an estimated population of 6,510.

**Figure 1:**
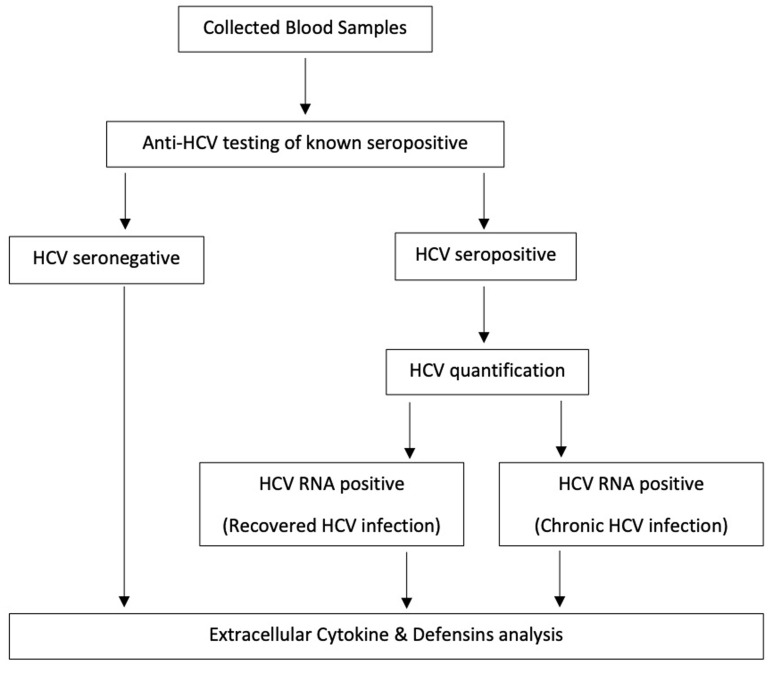
flow diagram of study laboratory investigations

**Data collection:** individuals who were rapidly diagnosed HCV seropositive and above 8 years were selected and recalled to participate in the current study. Subjects who have not yet initiated HCV treatment and voluntarily gave informed consent were enrolled. Persons who were HCV seronegative were also enrolled as controls. Persons who were ill or did not provide consent were excluded from the study. Individuals who gave informed consent were administered with questionnaires to obtain information on their socio-demographic characteristics such as age, gender and alcohol use. The research was performed in accordance with the Declaration of Helsinki. Ethics was approved by the Kwame Nkrumah University of Science and Technology and the Komfo Anokye Teaching Hospital research ethics review committee before commencement (reference; CHRPE/AP/134/13, CHRPE/AP/443/13 and CHRPE/AP/162/15). Permission was also taken from the study locations and written consent obtained from all participants. Confidentiality of participants information was maintained at each level of the study. Socio-demographic data were collected through face-face interview with participants in an enclosed area at a recruitment centre. Information was collected by trained research assistants. Socio-demographic study variables that were collected include gender, age, use of alcohol and hepatitis B status. Figure 1 shows the flow of laboratory investigations performed. Five millilitres of venous blood samples were collected from study participants into EDTA and serum vacutainers and transported at the required temperature (3°C to 6°C) to the laboratory until processing.

Samples were centrifuged at 3,000 rpm for 10 minutes and aliquots stored at -80°C until analysed. All study participants were re-tested for anti-HCV due to the relatively low specificity of the earlier test with rapid diagnostic testing kits. Processed blood samples were tested for HCV seropositivity using the assay: ORTHO® HCV version 3.0 ELISA Test System (Ortho Clinicals Incorporated, USA). Assay was performed according to the manufacturer´s protocol. Samples were added to microwell plates which has been precoated with recombinant hepatitis C encoded antigens c22-3, c200 and NS5 regions for the detection HCV specific antibodies. Samples that were confirmed to be positive for HCV antibodies were further tested for HCV RNA and quantified using the Abbott Real-Time RT-PCR (Abbott, IL, USA) Automated System. The assays were performed according to the manufacturer´s instructions and protocol. An Abbott m2000sp instrument is used with an Abbott mSample Preparation System to obtain HCV nucleic acids as well as stabilize them. Purified HCV RNA were amplified for detection using the Abbott Real-Time RT-PCR system and an amplification mastermix (HCV oligonucleotide reagent, thermostable rTth polymerase enzyme and activation reagent). Participants who tested sero-positive for HCV but viral RNA negative were classified as being spontaneously recovered from the HCV infection. Persons who tested sero-positive for HCV and also had detectable HCV RNA were classified as patients with active or chronic HCV infection [9].

Samples from all the study participants were analysed for the relative expressed levels of Th-1 cytokines; interleukin 2 (IL-2), interferon (IFN) gamma and tumour necrosis factor (TNF) alpha. Cytokines were quantitated using commercially available ELISA assay (Affymetrix eBioscience ELISA Ready-SET-Go, San Diego, California) according to the manufacturer´s instructions. High protein binding ELISA plates were coated with capture monoclonal antibody to detect and quantify the cytokines. Serial dilution of the assay´s concentrated standards were parallel analysed to quantify the concentrations from the samples. The plate wells were washed and further incubated with biotin-conjugated antibodies. Horseradish peroxidase-conjugated streptavidin was added to detect the listed cytokines. The optical density was determined using TECAN Sunrise ELISA reader (Tecan Group Ltd. Switzerland). The concentrations of the samples were determined using the standard (best-fit) curve plot. The serum levels of alpha and beta defensins were determined using commercially available ELISA assay for Human Defensin Beta 1 (HBD-1) and Human Alpha Defensins 1 (HAD-1) (SunLong Biotech Co. LTD, Hangzhou, China). The micro-well plates were pre-coated with an antibody specific to HBD-1 or HAD-1 (mouse monoclonal). The assay protocol was done according to the manufacturer´s instructions. The assay´s standards were prepared by 2-fold serial dilution and analysed alongside the test samples.

Samples and standards were added to pre-coated wells, incubated and washed. Horseradish peroxidase-conjugated streptavidin (conjugate) reagent was added to each well, incubated and unbounded excesses were washed. Substrate chromogen (0.34% 3,3',5,5'-tetramethylbenzidine) with 0.1% hydrogen peroxide were added to the wells and incubated away from light. The reaction was terminated by addition 1N H2SO4. The absorbance optical density of each well was read at 450nm using a micro-plate reader, TECAN Sunrise ELISA reader (Tecan Group Ltd. Switzerland). The concentrations of the samples were determined using the standard (best-fit) curve plotted using graphpad prism V.4.0 (GraphPad Software, San Diego, CA 92108 USA). All samples were serologically assessed for hepatitis B virus surface antibody (anti-HBsAg). The assays were done using GS HBsAg EIA 3.0 (BIO-RAD, Redmond, Washington, USA) and according to the manufacturers protocol. Serum samples were added to microwell plates which were precoated with antibody to HBsAg (mouse monoclonal) to detect specific human HBsAg antibodies. The serum levels of the liver enzymes alanine aminotransferase (ALT) and aspartate aminotransferase (AST) levels were measured using ALT/GPT 4+1 SL (ELITech Clinical Systems) on Selectra ProS automated chemistry analyser (ELITech Company, Japan) per protocol.

**Data analysis:** categorical variables were analysed using Chi-square test. Continuous variables were analysed using either parametric or non-parametric methods based on the distribution of the variables. Statistical analyses were performed using IBM SPSS Statistics v.12 (IBM Computer hardware company, New York, USA). From the analysis, p-value of ≤0.05 was considered statistically significant.

## Results

**Figure 2:**
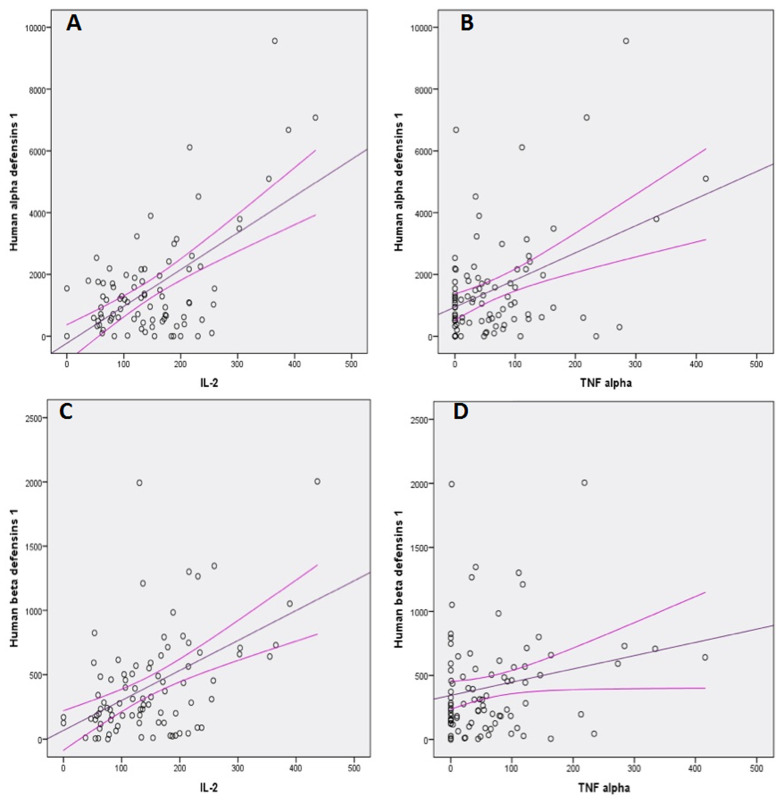
correlation of Cytokines: IL-2 and TNF with human alpha and beta defensins

A total of 132 persons participated in the study. Fifty-five were determined to have HCV chronic infection and 33 had spontaneously recovered from HCV infection. Forty-four anti-HCV negative were enrolled as HCV negative controls. All hundred and thirty-two samples were selected from the three study sites. Table 1 indicates 66% of the participants enrolled were male and majority had ages between 30 and 49 years (48.5%). Only 19.7% drank alcohol but 12.4% had hepatitis B viral co-infection. There was no association between the status of HCV and the gender, age of study participants and drinking alcohol. There was statistical significance difference in the status of HCV infection with and without hepatitis B co-infection (χ^2^= 6.5, df=2, P = 0.030). Persons with co-infection of hepatitis B are likely to develop chronic HCV infection. Liver enzymes alanine transaminase and aspartate transaminase were comparatively higher in the serum of subjects with chronic HCV infection, P = 0.014 and P = 0.000 respectively. Table 2 demonstrates the mean serum levels of the cytokines: IL-2, TNF alpha and IFN gamma was comparative among subjects with chronic, recovered and HCV negative groups. The mean levels of HAD-1 and HBD-1 was not significantly different among the various HCV infection statuses, P = 0.982 and 0.810 respectively. Figure 2 shows the Pearson correlation between serum Th-1 cytokines and human defensin. The levels of HAD-1 showed statistically significant correlation with IL-2 (r = 0.595, P = 0.000) and TNF alpha (r = 0.418, P = 0.000; Figure 2) but not IFN gamma (P = 0.367). HBD-1 only showed positive association with IL-2 (r = 0.491, P = 0.000) and not with TNF alpha (p = 0.052) and IFN gamma (p = 0.594) (Figure 2). The results of analysing HAD-1 and BD-1 among individuals with HCV chronic infection, those who have spontaneously recovered and HCV negative individuals suggest that IL-2 and TNF alpha could be associated with the activation or expression of HAD-1 and HBD-1.

**Table 1:** characteristics of study participants

Characteristics	HCV status			Total, n=132	P-value
	HCV chronic, n=55	HCV recovered, n=33	HCV negative, n=44		
**Gender**					**0.925**
Male	37(28%)	22(17%)	28(21%)	87(66%)	
Female	18(14%)	11(8%)	16(12%)	45(34%)	
**Age, y**					**0.771**
8-29	20(15.2%)	14(10.6%)	21(15.9%)	55(41.7%)	
30-49	28(21.2%)	16(12.1%)	20(15.2%)	64(48.5%)	
50-86	7(5.3%)	3(2.3%)	3(2.3%)	13(9.8%)	
**Drink alcohol**					**0.967**
Yes	11(8.3%)	6(4.5%)	9(6.8%)	26(19.7%)	
No	44(33.3%)	27(20.5%)	35(26.5%)	106(80.3%)	
**HBsAg status**					0.039*
Positive	10(7.8%)	5(3.9%)	1(0.8%)	16(12.4%)	
Negative	43(33.3%)	27(20.9%)	43(33.3%)	113(87.6)	
AST (mean, SD)	34.8(19.4%)	21.6(9.3%)	24.3(11.2%)	28.1(16.0)	0.000*
ALT (mean, SD)	38.7(31.1%)	23.8(17.1%)	28.1(17.5%)	31.5(24.9%)	0.014*

Comparison of HCV infection status determined by 2-tailed χ^2^; ^*^ Significant P-value <0.05

**Table 2:** HCV status and the levels of serum levels of cytokines and human defensins

Variables	HCV status, mean (SD)			P-value
	HCV chronic, n=55	HCV recovered, n=33	HCV negative, n=44	
**Cytokines**				
IL-2 (pg/mL)	153(91.6)	146(93.8)	144(70.2)	0.918
TNF (pg/mL)	56(72.0)	86(94.8)	54(68.0)	0.220
IFN (pg/mL)	187(288.8)	231(480.6)	241(443.1)	0.820
**Defensins**				
Human alpha defensins (pg/mL)	1377(1895.6)	1327(1446.5)	1309(1378.9)	0.982
Human beta defensins (pg/mL)	337(367.6)	392.9(400.3)	346(387.6)	0.810

No significant difference was observed in the levels of the immune markers (variables) among HCV chronic, recovered and negative. Significant χ^2^; P-value <0.05

## Discussion

From this study gender, age group and the use of alcohol did not show any significant association with HCV infection status. Similar to the present study. Some studies have suggested spontaneous HCV clearance among female, which could be attributed to the difference in hormonal levels since disease treatment response are significantly poor in menopausal women compared to reproductive aged women [10]. Consumption of alcohol has been thought to interfere with the rate of HCV clearance due to its immunosuppressive properties. However, our study could not establish any significant relationship between alcohol consumption and HCV status. The current study showed subjects with HCV and hepatitis B co-infection are likely to develop chronic HCV infection, positive for HCV RNA. The study results is similar to other studies which showed HCV-HBV co-infection leading to high viraemia with increased rates of cirrhosis and hepatocellular carcinoma [11]. AST and ALT levels were significantly high subjects who had chronic HCV infection; HCV RNA positive as compared with those who have recovered; HCV RNA negative and those who were anti-HCV negative. Park *et al*. observed a similar outcome in their study to assess the usefulness of this ratio as a predictor of cirrhosis [12]. Activated CD4+ T cells during viral infections differentiate into subsets of T cell helper effector cells. The interaction between T cells and hepatocytes is through the production of cytokines which influence the regulation of these lymphocytes and human defensins has also been found to induce cytokines, chemokines and activates TLR4 iDCs. In the current study, the serum levels of the Th-1 cytokines; IL-2, TNF alpha and IFN gamma was not different among the various groups of study subjects.

Other studies have showed notable increase in TNF alpha among children with chronic HCV infection as compared with healthy blood donors, whilst IL-2 was not detected in both groups [13]. Priimagi *et al*. found high serum concentrations of IFN-γ during chronic HCV infection whilst IL-2 was significantly decreased [4]. The variation might be due to the different sample population and processing that were used. IL-2 has been identified to promote the differentiation of specific immature T cells into effector and regulatory T cells whilst TNF-α is involved in the apoptotic signalling pathway of hepatocytes infected by HCV which could potentially decrease viral replication [14,15]. However, ours showed no significant effect of the levels of TNF alpha on HCV status. In the current study, human alpha 1 and human beta defensins 1 had no association with the status of HCV infection: chronic, recovered or anti-HCV negative. However, HCV core protein has been found to significantly activate in vitro the alpha-defensins transcription and invitro antiviral activity of human alpha and beta defensins against HCV includes complete neutralization of HCV particles as well as inhibition of viral entry into the cells and subsequent replication [16].

This study identified an association between HAD-1 and the levels of TNF alpha and IL-2 among the study participants. HBD-1 significantly had positive correlation with only IL-2. Another study indicated the presence of human beta defensins (HBDs) in human mast cell lines induced the release inflammatory mediators including IL-2 [17]. Signalling pathways initiated by human beta defensin-3 has been found to enhance IL-2 secretion from T cell receptor -activated T lymphoblast [6]. Some studies have identified increased secretion of IL-2 in specific target cells such as primary cultured conjunctival and lung cells when they were stimulated with both human alpha defensins, human beta defensins (HBD) 2, HBD-3 and human neutrophil peptides (HNP) respectively [18,19]. The cellular functional expression of human alpha defensins depend on effect and signalling of tumour necrosis factor receptors; keratinocytes stimulated with TNF-alpha has been found to induce human beta defensins (HBD-2 and HBD-3) by activating STAT-1 and NF-kappaB signalling [20]. But in this study the level of TNF alpha had no association with human beta defensins-1.

## Conclusion

In summary, our study demonstrates that serum levels of human alpha defensins 1, human beta defensins 1, IL-2, IFN gamma and TNF alpha have no significant association with having chronic, spontaneously recovering or being negative to HCV infection. Other observations described in this study provide a significant positive correlation between the defensins studied and IL-2 cytokines during HCV infection. These finding suggest that inflammatory response through the production of cytokines IL-2 and TNF alpha by cells affect the release of human alpha and beta defensins. The serum concentrations of both defensins do not vary in patients with chronic HCV infection, spontaneously recovered or persons who are HCV negative. Further experimental studies could build up on this work to investigate the synergistic effect of human defensins and IL-2 in HCV recovery.

### What is known about this topic

- Human defensins (HAB-1, HDB-1) has been determined to have some anti-viral properties;
- The expression of T helper-1 cytokines, including IL-2, TNF alpha and IFN gamma is known to promote complete recovery from acute HCV infection.

### What this study adds

- This current study showed no significant relationship between the state of an HCV infection and the serum levels of HAD-1, HBD-1, IL-2, IFN gamma and TNF alpha;
- The current study however found a strong correlation between human defensins and IL-2 which could possibly indicate active release of IL-2 and TNF alpha by cells affect the production of human alpha and beta defensins;
- The study also confirmed the important role of serum AST and ALT markers in predicting chronic HCV infection.
